# Ginkgolide injections in meglumine, combined with edaravone, significantly increases the efficacy in acute ischemic stroke: A meta-analysis

**DOI:** 10.3389/fphar.2023.1236684

**Published:** 2024-04-25

**Authors:** Mingyuan Yan, Jing Wu, Le Wang, Kaiyue Wang, Lili Li, Tianye Sun, Han Zhang, Mi Zhang, Lin Zou, Songyi Yang, Jinmin Liu

**Affiliations:** ^1^ Beijing University of Chinese Medicine, Beijing, China; ^2^ Dongzhimen Hospital, University of Chinese Medicine, Beijing, China; ^3^ Encephalopathy Department I, Dongfang Hospital, Beijing University of Chinese Medicine, Beijing, China

**Keywords:** diterpene ginkgolides meglumine injection, traditional Chinese medicine, acute ischemic stroke, NIHSS, meta-analysis

## Abstract

**Objective:**

This study aimed to evaluate the efficacy of combining diterpene ginkgolide meglumine injection (DGMI) with edaravone for the treatment of acute ischemic stroke. This is particularly relevant because Western drugs, excluding intravenous thrombolysis, have shown limited success.

**Methods:**

A comprehensive search was conducted using multiple databases, including PubMed, Cochrane Library, Web of Science, China National Knowledge Infrastructure WanFang, VIP, and Chinese Biomedical Database (CBM) until June 2023. The data were analyzed using fixed-effects and random-effects models in Review Manager. The mean difference with 95% confidence interval was calculated for each outcome.

**Results:**

Eighteen studies involving 1,636 participants were included in the analysis. The DGMI group showed significant reductions in the National Institutes of Health Stroke Scale (NIHSS) score, modified Rankin Scale (mRS) score, and C-reactive protein (CRP) level, compared to the control group. Furthermore, the DGMI group showed a significant improvement in superoxide dismutase (SOD) levels and a reduction in malondialdehyde (MDA) levels. The combination of DGMI and edaravone was more effective in reducing neuron-specific enolase (NSE) levels following brain tissue injury than edaravone alone. Additionally, DGMI complemented edaravone in reducing rheological parameters associated with ischemic stroke, including hematocrit, plasma viscosity, platelet adhesion rate, and erythrocyte deformation index.

**Conclusion:**

The combination of DGMI and edaravone significantly improved the therapeutic efficacy in patients with acute ischemic stroke. However, more extensive and high-quality clinical trials are required to validate these underlying mechanisms.

**Systematic Review Registration:**

https://www.crd.york.ac.uk/PROSPERO/display_record.php?RecordID=260215, identifier: PROSPERO (CRD42021260215)

## Introduction

Acute ischemic stroke occurs when the blood supply to the brain is suddenly blocked or severely reduced, leading to ischemia, hypoxic necrosis, and softening of local brain tissue, resulting in rapid loss of brain function in the affected area. Ischemia-induced stroke is responsible for 68% of all strokes worldwide, ranking as the second leading cause of death and the third leading cause of disability. Despite primary prevention efforts, the annual incidence of stroke has continued to increase (GBD, 2019 Stroke Collaborators, 2021). China has the highest global incidence and death rate of stroke (Feigin et al., 2014; Wang et al., 2017; Song et al., 2019), posing a significant and urgent public health concern. Prompt revascularization of the occluded blood vessels is crucial for stroke management. Revascularization therapies, including intravenous thrombolysis, intravascular mechanical thrombus retrieval, and antiplatelet therapy, are effective interventions for improving cerebral infarction and are recommended by the Chinese Medical Association guidelines for ischemic stroke (Peng and Wu, 2018). However, the use of intravenous thrombolysis is limited owing to time constraints, stringent criteria, and limited hospital accessibility, among other factors. Traditional Chinese Medicine (TCM) plays an important role in the treatment of various diseases. The modernization of Chinese medicine has established an effective stroke treatment system that includes the use of herbal extracts. Given the limited success of Western medicine alone in acute ischemic stroke patients who cannot undergo thrombolysis, Chinese doctors have traditionally used a combination of Western and Chinese medicine (Gao et al., 2017). This combination of Western medicine and Chinese herbal extracts has proven beneficial for many stroke patients, particularly in improving brain microcirculation, reducing inflammation and edema, and providing antioxidant effects.

The diterpene ginkgolide meglumine injection (DGMI) is a Chinese medicinal product derived from *Ginkgo biloba* extract. The primary constituents of this drug are ginkgolides A, B, and K. To ensure a rigorous scientific conclusion, we referred to the ConPhyMP statement, which provides a comprehensive classification of drugs derived from plant extracts (Heinrich et al., 2022). The name of *G. biloba* has been verified in two databases (Plant of the World Online and The World Flora Online). They belong to a category of drugs that enhance blood circulation and resolve blood stasis. According to this statement, drugs composed of plant extracts can be categorized into three types, and DGMI meets the criteria for Type A extraction. DGMI is derived from the leaf extract of *G. biloba*, a plant belonging to the Ginkgoaceae family. Traditional medicine has been extensively researched and developed, revealing its potent antioxidant and anti-free-radical properties. The active components of DGMI are GA, GB, and GK, which are present at a ratio of 15:30:1 in this injection. Meglumine serves as a co-solvent in this injection, without contributing to its therapeutic effect. The intellectual property of the DGMI is exclusively owned and produced by Kanion Pharmaceutical, a Chinese company. Currently, the drugs used in all hospitals in China are uniform and adhere to certain specifications. Each bottle contains 5 mL of an injection with *G. biloba* diterpenoid lactone (25 mg and is administered as an injection. This TCM was approved by the SFDA on 30 October 2012, with approval number Z20120024. To minimize heterogeneity, all studies included in the analysis used the DGMI produced by the same manufacturer. More information about this drug has been listed in Table 1.

**TABLE 1:** Detailed information of DMGI accroding to the ConPhyMP statement.

Injection name	Injection sourse	Botanical plant name	Species	Plant part used	Origin and harvest time	Extraction and production methods
Diterpene ginkgolide meglumine injection	Jiangsu Kanion Pharmaceutical Co., Ltd	Ginkgo biloba L	Ginkgoaceae	Dry leaves of Ginkgo biloba L	China. Autumn	Under patent protection, we are unable to obtain specific information about the extraction and production process

Drug efficacy in TCM	Functional Indications	Quality control reported?	Chemical analysis report?	Injection composition	Proportion of main components and excipients	Drug properties
Promoting blood circulation to remove meridian obstruction	Used for cerebral infarction, symptoms such as hemiplegia, crooked tongue, awkward speech, numbness of limbsetc.	Yes. Z20120024, approved by China Foods Limited and Drug Administration	No.	Main components: Ginkgolides A, Ginkgolides B, Ginkgolides K(15:30:1). Excipients: Meglumine, Citric Acid, Sodium chloride	98:2	Colorless to yellowish clear liquid
Adverse reactions						
1. Allergic reactions: chills, fever, panic, discomfort in the back pillow, slight cyanosis of the lips and claws, shaking of the lower limbs, skin rash, flushing, Telangiectasia, sweating, skin itching and chest tightness, cough, nasal congestion and runny nose, etc. 2. Systemic damage: fever, back pain, neck distension, discomfort in the occipital region. 3. Respiratory system: coughing, stuffy and runny nose, chest tightness, chest discomfort. 4. Cardiovascular system: blood pressure fluctuations mainly include blood pressure reduction, palpitation, palpitation, supraventricular premature contraction, complete or incomplete Right bundle branch block, first degree atrioventricular block, atrial fibrillation and other ECG abnormalities. 5. Neuropsychiatric system: dizziness, headache, drowsiness, insomnia, lower limb shaking, abnormal coordination function. 6. Digestive system: nausea, vomiting, loss of appetite, abdominal discomfort, diarrhea, positive fecal occult blood, alanine aminotransferase, Asparagus cochinchinensis aminotransferase, Alkaline phosphatase and γ- Glutamic transferase, single or multiple elevated. 7. Blood system: thrombocytopenia, red blood cell decline, hemoglobin decline, Prothrombin time extension, activated partial thromboplastin time extension, fibrinogen reduction. 8. Skin and its accessories: Phlebitis and ecchymosis at the infusion site. 9. Kidney and Urinary system: excessive urination, increased nocturnal urine, abnormal blood creatinine, abnormal Blood urea nitrogen, positive urine red blood cells, positive urine protein, positive urine sugar						

Modern medicine acknowledges the potential of these drugs to improve cerebral blood circulation, increase blood flow, and prevent platelet aggregation (Zhang Q. et al., 2021; Sun et al., 2021). DGMI is typically used in the treatment of cerebral infarction, often in combination with Western drugs, resulting in superior outcomes compared with Western drugs alone. These benefits are evident in NIHSS scores, blood rheological indicators, and other serum indicators (Zhang et al., 2017; Li, 2019; Liu et al., 2019; Wang, 2019). Moreover, animal studies have suggested that ginkgolides A, B, and K have potential as PAFR antagonists, offering unique advantages in protecting the blood-brain barrier, reducing brain edema, and exhibiting antioxidant effects. These mechanisms involve regulation of inflammatory molecule release, vascular endothelial growth factor expression, and neurogenesis (Wang et al., 2007; Liu et al., 2010; Ma et al., 2012; Nabavi et al., 2015; Liu et al., 2017; Zhong, 2020; Yang et al., 2021). Although several clinical studies have reported the efficacy of DGMI in combination with Western drugs for the treatment of cerebral infarction, these studies used different Western drugs, and there has been no systematic evaluation or analysis. Therefore, to minimize bias and provide evidence-based treatment for ischemic stroke using DGMI, we selected edaravone as the preferred Western drug in our study.

## Methods

### Systematic review protocol and registration

This study followed the guidelines outlined in the Preferred Reporting Items for Systematic Reviews and Meta-Analyses (PRISMA) statement (Moher et al., 2009) and the methodology recommended by the Cochrane Handbook for Systematic Reviews of Interventions. The protocol for this study was registered in PROSPERO (CRD42021260215).

### Search strategy

**FIGURE 1:**
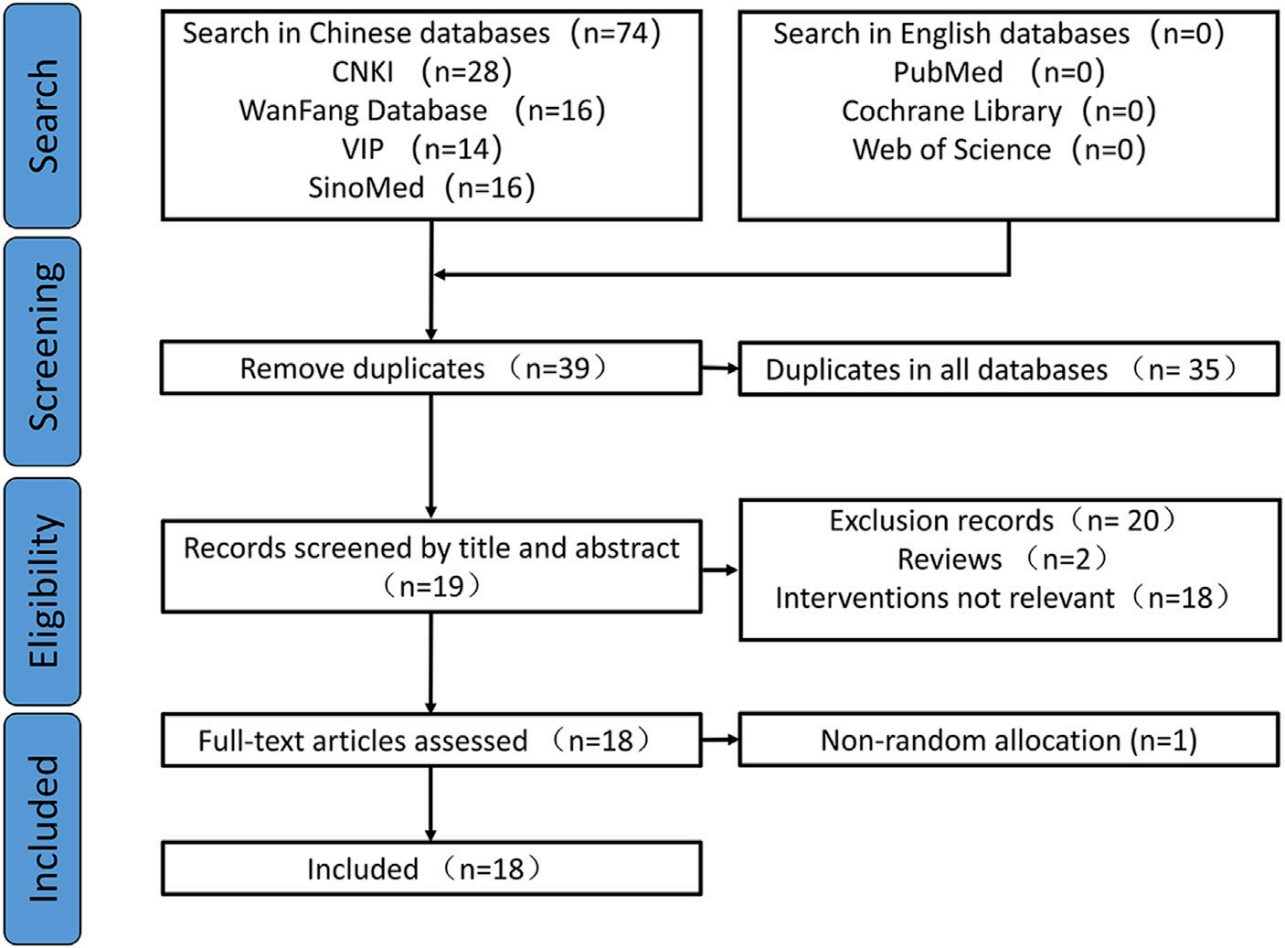
Flowchart for study screening.

A comprehensive search was conducted in three English databases (PubMed, Cochrane Library, and Web of Science) and four Chinese databases (CNKI, WanFang, VIP, and CBM) until June 2023. The databases were searched using terms such as stroke, ischemic stroke, cerebral infarction, diterpene ginkgolide meglumine injection, *Ginkgo* diterpene lactone meglumine, *G. biloba* diterpene lactone glucosamine, and randomized controlled trials. The search encompassed both electronic and manual sources of information. The identified studies were compiled, and duplicates, animal experiments, reviews, studies with unextractable data, and studies that could not be systematically evaluated were excluded, resulting in the selection of studies for further analysis. The screening process for these studies is depicted in Figure 1.

### Eligibility criteria

The inclusion criteria were as follows: 1) Participants who met the diagnostic criteria for acute ischemic stroke and exhibited significant symptoms. 2) Patients aged between 18 and 90 years in whom the time from onset to consultation did not exceed 72 h. 3) The included literature reported at least the NIHSS score as an outcome indicator. 4) The study was approved by the ethics committees of the participating hospitals. 5) The study design was a randomized controlled trial. 6) The control group intervention involved edaravone, whereas the experimental group intervention involved a combination of edaravone and diterpene ginkgolide meglumine injection. 7) Studies limited to English and Chinese languages.

The exclusion criteria were as follows: 1) Duplicate publications, animal studies, and reviews. 2) Studies that did not align with the diagnosis of acute ischemic stroke, including transient ischemic attack. 3) Studies with incomplete data on relevant outcome indicators.

### Study selection and data extraction

Two researchers (MY Yan and J Wu) independently screened the titles and abstracts of the studies based on the inclusion and exclusion criteria. The investigators compared their screening results and consulted a third person (L Wang) for a final decision in case of disagreement. Data extraction involved collecting information, such as the first author’s name, year of publication, study design, participant characteristics, interventions, control measures, duration of intervention, outcome indicators, adverse effects, and continuous data are presented as mean ± standard deviation.

### Assessment of risk of bias in individual studies

**FIGURE 2:**
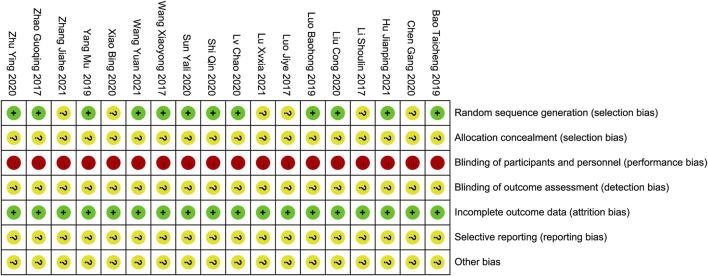
Risk of bias summary.

**FIGURE 3:**
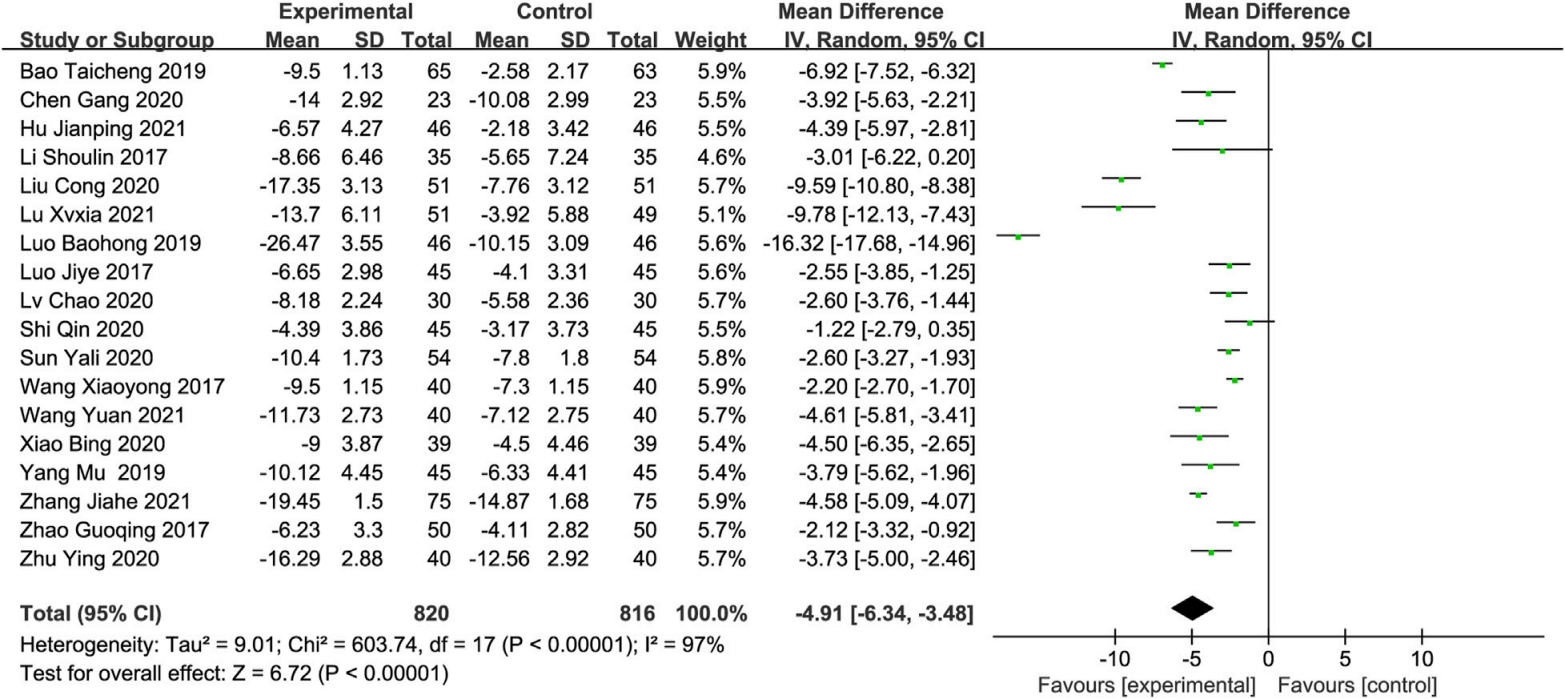
Risk of bias graph.

The studies were assessed for quality using the Cochrane “Bias Risk Assessment Tool,” (Higgins et al., 2011) which evaluated randomization methods, allocation concealment, blinding, completeness of outcome data, selective reporting, and other biases. Other biases were identified by thoroughly analyzing the articles and assessing the adequacy of baseline characteristics and outcome indicators. The evaluations were categorized as “low risk of bias”, “high risk of bias,” or “unclear.” All included studies acknowledged random allocation, with ten reporting the use of a random number table, one reporting a random lottery, and one using a random coin flip, with a minimal risk of bias. The method of randomization in the six remaining studies was unclear, resulting in an uncertain risk of bias. Allocation concealment was not addressed in these studies, resulting in a risk of bias. All studies had a high risk of performance bias owing to a lack of blinding reporting. Insufficient data hindered the evaluation of additional biases, resulting in uncertain risks. All the included studies were assessed as having a low risk of incomplete outcome data. The risk of selective reporting in these studies could not be determined due to insufficient evidence. These trials did not provide information on data management, statistical strategies, or implementation processes, resulting in ambiguous risk assessments. Based on these factors, it is reasonable to assume that all the included studies were of low quality. The quality assessment of the studies is presented in Figures 2, 3.

### Statistical analysis

Statistical analyses were conducted using Review Manager 5.3 (Cochrane Collaboration, Copenhagen, Denmark). We estimated the combined effects of the interventions on continuous outcomes using the mean differences(MDs) with 95% confidence intervals (CIs). Meta-analyses were performed using Mantel-Haenszel fixed-effects models in the absence of significant statistical heterogeneity among the included studies. A significance level of 5% was used throughout the study. Heterogeneity between studies was assessed using the Q and I^2^ methods. A *p*-value >0.1 and an I^2^ value <50% indicated homogeneity in the literature and were analyzed using a fixed-effects model. Conversely, if heterogeneity was detected (*p*-value ≤0.1 or, I^2^ ≥ 50%), a random-effects model was used. Sensitivity tests and subgroup analyses were performed to investigate the possible causes of heterogeneity.

## Outcomes

The primary outcome indicator was the National Institute of Health Stroke Scale (NIHSS), while the additional outcome indicators included the Barthel index (BI), neuron-specific enolase (NSE), C-reactive protein (CRP), malondialdehyde (MDA), superoxide dismutase (SOD), modified Rankin scale (mRS), and hemorheological indices such as hematocrit, plasma viscosity (PV), erythrocyte deformation index, and platelet adhesion rate.

## Results

### Compilation of studies and characteristics

A total of 76 Chinese studies were initially retrieved. Through a rigorous process involving the careful evaluation of titles and abstracts and thorough screening by two reviewers, 18 randomized controlled trials (Li et al., 2017a; Luo et al., 2017; Zhao et al., 2017; Bao and Peng, 2019; Luo and Chen, 2019; Yang, 2019; Chen et al., 2020; Liu, 2020; Lv et al., 2020; Shi and Fan, 2020; Sun, 2020; Xiao, 2020; Zhu, 2020; Zhang et al., 2021b; Wang et al. 2024; Hu et al., 2021; Lu, 2021; Wang and Fan, 2021) conducted in China were selected for this meta-analysis. The analyzed studies were published between 2017 and 2021 and included 1,636 participants, with 820 assigned to the control group and 816 assigned to the experimental group. All studies reported NIHSS scores, and four studies (Luo et al., 2017; Luo and Chen, 2019; Hu et al., 2021; Wang et al. 2024) included data on BI post-treatment. Four studies reported NSE levels (Li et al., 2017a; Luo and Chen, 2019; Yang, 2019; Zhang et al., 2021b), while three studies reported CRP levels (Sun, 2020; Zhang et al., 2021b; Wang and Fan, 2021). Two studies reported MDA and SOD levels (Sun, 2020; Wang and Fan, 2021), and three studies reported hemorheological indices, including hematocrit, plasma viscosity, erythrocyte deformation index, and platelet adhesion rate (Chen et al., 2020; Zhang et al., 2021b; Lu, 2021). Two studies reported the mRS scores (Bao and Peng, 2019; Wang et al. 2024). Basic information and details of the included studies are presented in Tables 2 and 3, respectively.

**TABLE 2:** Basic characteristics of the included studies.

Study	Sample size E C	Age (years) E/C	Male/female E C	Population	Interventions E/C	Duration
Luo et al. Luo and Chen, (2019)	46 46	58.62 ± 5.14/58.58 ± 5.11	20/26 21/25	AIS within 6 h	DGMI + edaravone/edaravone	14 d
Li et al. Li et al. (2017a)	35 35	66.40 ± 7.65/65.31 ± 7.81	20/15 23/12	AIS within 48 h	DGMI + edaravone + RT/edaravone + RT	14 d
Xiao et al. Xiao, (2020)	39 39	65.2 ± 6.5/66.0 ± 6.0	24/15 22/17	AIS within 48 h	DGMI + edaravone + RT/edaravone + RT	14 d
Shi et al. Shi and Fan, (2020)	45 45	69.5 ± 4.8/68.4 ± 5.1	28/17 26/19	AIS	DGMI + edaravone + RT/edaravone + RT	14 d
Lv et al. Lv et al. (2020)	30 30	62.20 ± 5.41/62.15 ± 5.36	17/13 18/12	AIS within 24 h	DGMI + edaravone + RT/edaravone + RT	14 d
Hu et al. Hu et al. (2021)	46 46	62.27 ± 3.68/62.35 ± 3.59	35/11 37/9	AIS within 6 h	DGMI + edaravone + RT/edaravone + RT	28 d
Liu et al. Liu, (2020)	51 51	60.62 ± 6.54/60.59 ± 6.51	31/20 30/21	AIS within 7 h	DGMI + edaravone/edaravone	14 d
Sun et al. Sun, (2020)	54 54	60 ± 6/60 ± 6	32/22 30/24	AIS within 24 h	DGMI + edaravone/edaravone	14 d
Yang et al. Yang, (2019)	45 45	53.04 ± 4.02/51.79 ± 4.33	25/20 29/16	AIS within 48 h	DGMI + edaravone + RT/edaravone + RT	14 d
Zhao et al. Zhao et al. (2017)	50 50	52.19 ± 2.28/53.18 ± 2.16	28/22 24/26	AIS within 7d	DGMI + edaravone/edaravone	14 d
Chen et al. Chen et al. (2020)	23 23	51.49 ± 2.71/51.57 ± 2.19	14/9 13/10	AIS within 6 h	DGMI + edaravone + RT/edaravone + RT	14 d
Zhu et al. Zhu, (2020)	40 40	64.29 ± 9.7/63.65 ± 9.3	21/19 22/18	AIS	DGMI + edaravone/edaravone	14 d
Bao et al. Bao and Peng, (2019)	65 63	64.50 ± 5.50/66.50 ± 5.00	30/35 30/33	AIS within 12 h	DGMI + edaravone + RT/edaravone + RT	14 d
Lu et al. Lu, (2021)	51 49	59.85 ± 10.24/60.02 ± 10.31	26/25 27/22	AIS	DGMI + edaravone + RT/edaravone + RT	NR
Zhang et al. Zhang et al. (2021b)	75 75	63.29 ± 3.62/62.38 ± 3.47	42/33 41/34	AIS within 6 h	DGMI + edaravone + RT/edaravone + RT	14 d
Wang et al. Wang et al. (2024)	40 40	75.5 ± 5.25/76.6 ± 5.25	22/18 19/21	AIS within 72 h	DGMI + edaravone + RT/edaravone + RT	14 d
Wang et al. Wang and Fan, (2021)	40 40	56.12 ± 1.42/55.86 ± 1.37	25/15 23/17	AIS within 48 h	DGMI + edaravone + RT/edaravone + RT	14 d
Luo et al. Luo et al. (2017)	45 45	75.46 ± 5.83/73.54 ± 6.32	27/18 28/17	AIS within 48 h	DGMI + edaravone + RT/edaravone + RT	14 d

**RT**: routine treatment; **AIS**: acute ischemic stroke; **E**: experimental group; **C**: control group; **DGMI**: diterpene ginkgolides meglumine injection; **NR**: not report.

**TABLE 3:** Additional information on the included studies.

Study	Drug dosage		Outcomes	Adverse effects
	DGMI	Edaravone		E/C
Luo et al. Luo and Chen, (2019)	25 mg + 250mL0.9%NS ivgtt qd	30 mg + 100mL0.9%NS ivgtt qd	effectiveness, NIHSS	1 case of diarrhea, 1 case of rash/1 case of diarrhea, 1 case of rash, 1 case of nausea
Li et al. Li et al. (2017a)	25 mg + 250mL0.9%NS ivgtt qd	30 mg + 100mL0.9%NS ivgtt bid	effectiveness, NIHSS	no adverse effects occurred
Xiao et al. Xiao, (2020)	25 mg + 250mL0.9%NS ivgtt qd	30 mg + 100mL0.9%NS ivgtt bid	effectiveness, NIHSS	not report
Shi et al. Shi and Fan, (2020)	25 mg ivgtt qd	30 mg ivgtt bid	NIHSS,Lp-PLA2,NSE,GFAP	not report
Lv et al. Lv et al. (2020)	25 mg + 250mL0.9%NS ivgtt qd	30 mg + 250mL0.9%NS ivgtt bid	NIHSS,ADL	not report
Hu et al. Hu et al. (2021)	25 mg ivgtt qd	30 mg ivgtt qd	NIHSS,FAM,BI,GABA,Gly,Hcy,NE,MPO,TNF	3 cases of cerebral vasospasm, 1 case of hydrocephalus, and 1 case of subcutaneous hemorrhage/cerebral vasospasm 10 cases, hydrocephalus 4 cases, subcutaneous hemorrhage 4 cases hydrocephalus 4 cases, subcutaneous hemorrhage 4 cases
Liu et al. Liu, (2020)	25 mg + 250mL0.9%NS ivgtt qd	30 mg + 150mL0.9%NS ivgtt qd	NIHSS, effectiveness,PV	not report
Sun et al. Sun, (2020)	25 mg + 250mL0.9%NS ivgtt qd	15 mg ivgtt qd	NIHSS,SOD,MDA,IL-6,CRP	not report
Yang et al. Yang, (2019)	25 mg + 250mL0.9%NS ivgtt qd	30 mg + 100mL0.9%NS ivgtt bid	NIHSS,Hcy,NSE	not report
Zhao et al. Zhao et al. (2017)	25 mg + 250mL0.9%NS ivgtt qd	30 mg + 100mL0.9%NS ivgtt qd	NIHSS, effectiveness	not report
Chen et al. Chen et al. (2020)	25 mg + 250mL0.9%NS ivgtt qd	30 mg + 100mL0.9%NS ivgtt bid	NIHSS,ADL,hematocrit,PV, erythrocyte deformation index, platelet adhesion rate	not report
Zhu et al. Zhu, (2020)	25 mg + 250mL0.9%NS ivgtt qd	30 mg + 100mL0.9%NS ivgtt bid	NIHSS	not report
Bao et al. Bao and Peng, (2019)	20 mg + 250mL0.9%NS ivgtt qd	30 mg + 100mL0.9%NS ivgtt bid	NIHSS,mRS	not report
Lu et al. Lu, (2021)	25 mg + 250mL0.9%NS ivgtt qd	30 mg ivgtt bid	NIHSS,FAM,MMSE, effectiveness,hematocrit, platelet adhesion rate,PV	1 case of headache, 1 case of nausea, 1 case of diarrhea, 1 case of chills/1 case of fever, 1 case of chest tightness, 1 case of rash, 1 case of dyspnea, 1 case of palpitations
Zhang et al. Zhang et al. (2021b)	25 mg + 250mL0.9%NS ivgtt qd	30 mg + 100mL0.9%NS ivgtt bid	NIHSS,BI,QOLISP,NSE,CRP,hematocrit,PV, erythrocyte deformation index, platelet adhesion rate	not report
Wang et al. Wang et al. (2024)	25 mg + 250mL0.9%NS ivgtt qd	30 mg + 100mL0.9%NS ivgtt bid	NIHSS,mRS,effectiveness	no adverse effects occurred
Wang et al. Wang and Fan, (2021)	25 mg + 250mL0.9%NS ivgtt qd	30 mg + 100mL0.9%NS ivgtt bid	NIHSS,BI,SOD,MDA,CRP,effectiveness	not report
Luo et al. Luo et al. (2017)	25 mg + 100mL0.9%NS ivgtt qd	30 mg + 100mL0.9%NS ivgtt bid	NIHSS,BI,hs-CRP,NSE,effectiveness	not report

**NS**: normal saline; **Lp-PLA2**: lipoprotein-associated phospholipaseA2; **CRP**: C-reactive protein; **GABA**: gamma-aminobutyric acid; **Gly**: glycine; **Hcy**: homocysteine; **NE**: noradrenaline; **MPO**: myeloperoxidase; **TNF**: tumor necrosis factor-α; **PV**: plasma viscosity; **BI**: barthel index; **NSE**: neuron-specific enolase; **GFAP**: glial fibrillary acidic protein; **SOD**: superoxide dismutase; **IL-6**: Interleukin-6; **mRS**: modified Rankin scale; **hs-CRP**: high sensitivity C-reactive protein; **QOLISP**: quality of life inventory for stroke patients; **MMSE**: Mini-mental State Examination; **DGMI**: diterpene ginkgolides meglumine injection; **MDA**: Malondialdehyde.

### Outcomes measurements

#### The National Institutes of Health Stroke Scalev (NIHSS)

**FIGURE 4:**
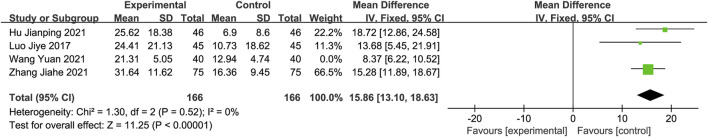
Forest plot of NIHSS scores.

All included studies (1,636 participants) reported the NIHSS score as an outcome measure. The meta-analysis found that the combination of DGMI and edaravone was more effective in reducing NIHSS scores than edaravone alone, based on the random-effects model (MD: −4.91; 95% CI: −6.34, −3.48; *p* < 0.00001, Figure 4). The studies showed significant heterogeneity (Chi^2^ = 603.74; I^2^ = 97%; *p* < 0.00001), and the cause of this heterogeneity could not be determined using the sensitivity analysis.

#### Barthel Index (BI)

**FIGURE 5:**
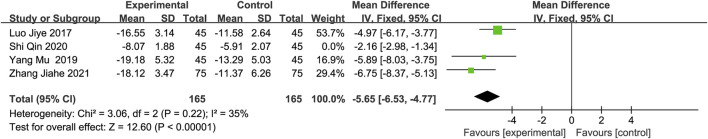
Forest plot of BI after excluding Wang’s study.

Only four studies (Luo and Chen, 2019; Zhang et al., 2021b; Hu et al., 2021; Wang and Fan, 2021), with 206 participants in each group, reported the BI score as an outcome measure. The meta-analysis demonstrated that the experimental group showed significantly greater improvement in BI than the control group, using a random-effects model. Sensitivity analysis revealed that the study conducted by Wang et al. (Wang and Fan, 2021) significantly influenced the heterogeneity of this group. After excluding this study, the heterogeneity disappeared. The results were then analyzed using a fixed-effects model (MD: 15.86; 95% CI: 13.10, 18.63; *p* < 0.00001). Heterogeneity was assessed using Chi^2^ and I^2^ statistics, which showed no significant heterogeneity (Chi^2^ = 1.30; I^2^ = 0%; *p* = 0.52, Figure 5).

#### Neuron-specific enolase (NSE)

**FIGURE 6:**
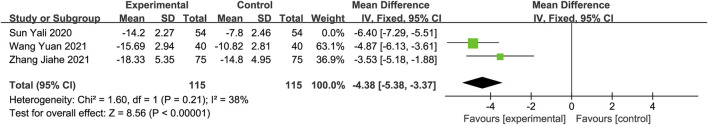
Forest plot of NSE after excluding Shi’s study.

Four studies (Luo and Chen, 2019; Yang, 2019; Shi and Fan, 2020; Zhang et al., 2021b) with 210 participants in each group reported NSE levels as an outcome measure. The random-effects model indicated that DGMI effectively reduced NSE levels. However, sensitivity analysis revealed that the study conducted by Shi et al. (Shi and Fan, 2020) had a significant effect on heterogeneity. After excluding this study, the heterogeneity decreased. A meta-analysis using a fixed-effects model confirmed this conclusion (MD: −5.65; 95% CI: −6.53, −4.77; *p* < 0.00001; heterogeneity: Chi^2^ = 3.06; I^2^ = 35%; *p* = 0.22, Figure 6).

#### C-reactive protein (CRP)

**FIGURE 7:**
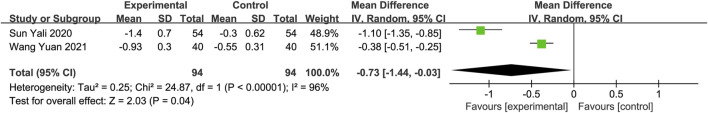
Forest plot of CRP after excluding Sun’s study.

CRP levels were reported in three studies (Sun, 2020; Zhang et al., 2021b; Wang and Fan, 2021) involving 338 participants. Meta-analysis using a random-effects model demonstrated a significant reduction in CRP levels in the experimental group compared to the control group. The sensitivity analysis identified the study conducted by Sun et al. (Sun, 2020) as a significant contributor to heterogeneity. After excluding this study, residual heterogeneity remained within an acceptable range. Therefore, a fixed-effects model was used for the analysis, resulting in the same conclusion (MD: −4.38; 95% CI: −5.38, −3.37; *p* < 0.00001; heterogeneity: Chi^2^ = 1.60; I^2^ = 38%; *p* = 0.21, Figure 7).

#### Malondialdehyde (MDA) and superoxide dismutase (SOD)

**FIGURE 8:**
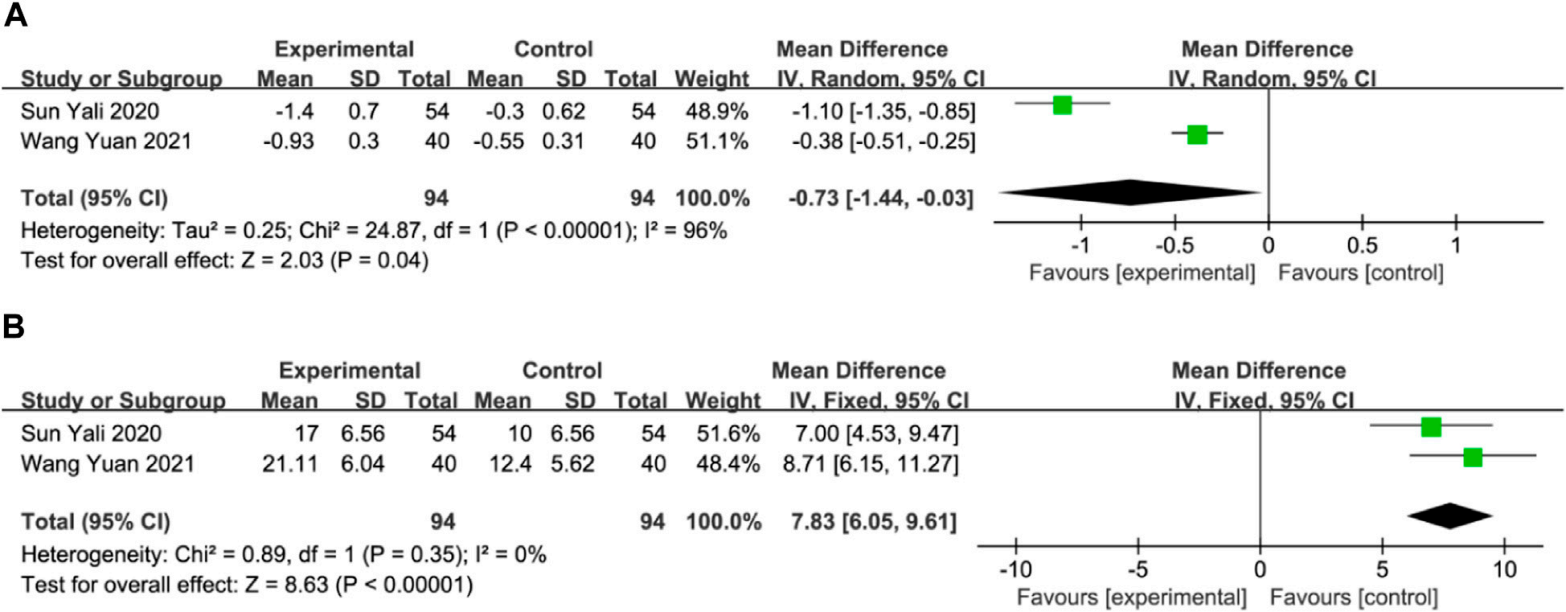
Forest plot of MDA **(A)** and SOD **(B)**.

Two studies with a total of 188 participants assessed MDA and SOD levels as outcome measures (Sun, 2020; Wang and Fan, 2021). The random-effects model revealed a significant reduction in MDA in the DGMI group compared to that in the control group (MD: −0.73; 95% CI: −1.44, −0.03; *p* = 0.04; heterogeneity: Chi^2^ = 24.87; I^2^ = 96%; *p* < 0.00001, Figure 8A). No heterogeneity was observed in the two studies regarding SOD. A fixed-effects model was used for the meta-analysis, which demonstrated a significant improvement in SOD levels in the experimental group compared with the control group (MD: 7.83; 95% CI: 6.05, 9.61; *p* < 0.00001; heterogeneity: Chi^2^ = 0.89; I^2^ = 0%; *p* = 0.35, Figure 8B).

### Blood Rheology Indices

**FIGURE 9:**
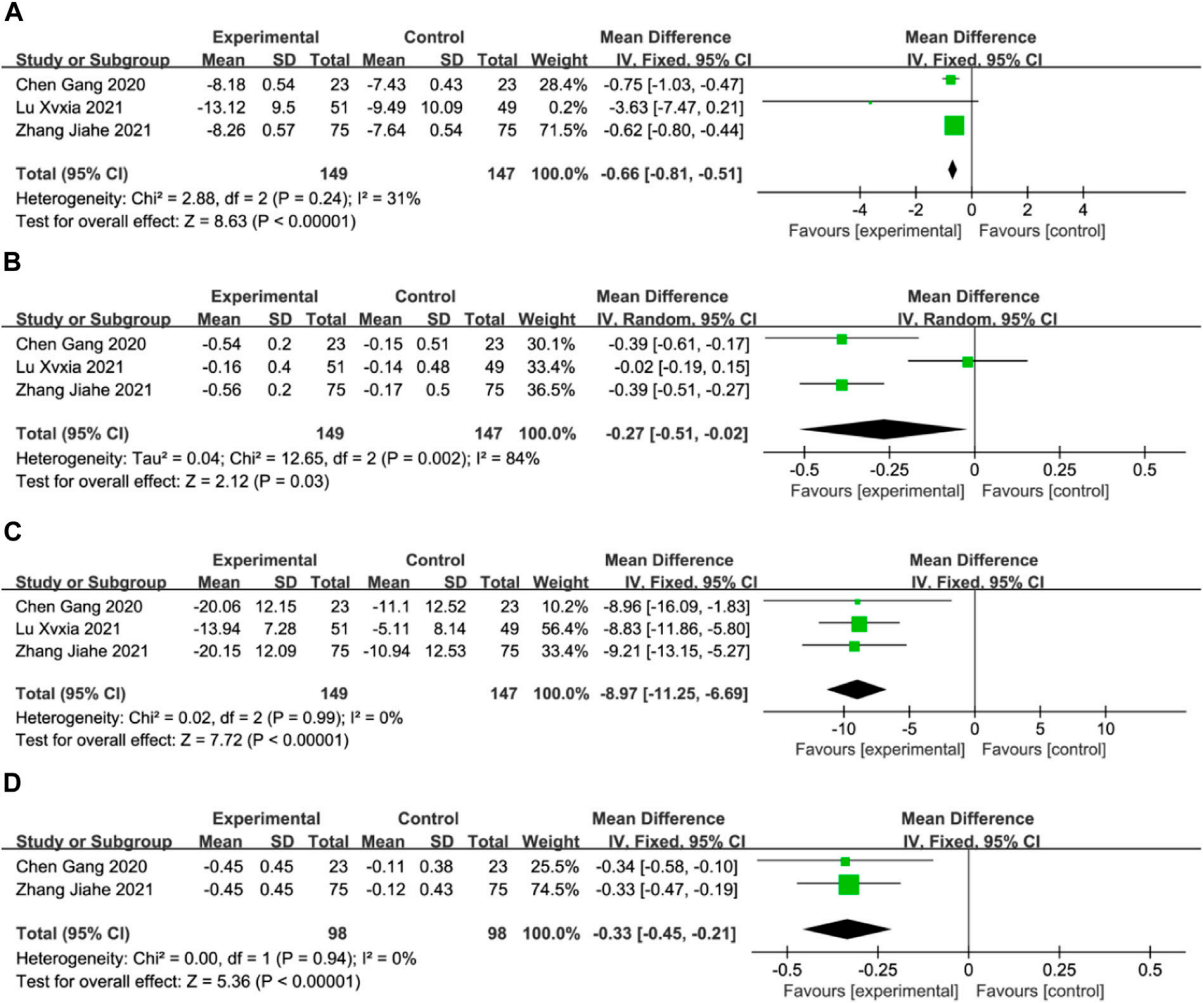
Forest plot of Blood Rheology Indices (A: forest plot of hematocrit, **(B)** forest plot of plasma viscosity, **(C)** forest plot of platelet adhesion rate, **(D)** forest plot of platelet erythrocyte deformation index).

Three studies reported the hematocrit, plasma viscosity, platelet adhesion rate, and erythrocyte deformation index (Chen et al., 2020; Zhang et al., 2021b; Lu, 2021), whereas two studies reported the erythrocyte deformation index (Chen et al., 2020; Zhang et al., 2021b). Meta-analysis using a fixed-effects model revealed that the DGMI group was more effective than edaravone alone in reducing the hematocrit (MD: −0.66; 95% CI: −0.81, −0.51; *p* < 0.00001; heterogeneity: Chi^2^ = 2.88; I^2^ = 31%; *p* = 0.24, Figure 9A), platelet adhesion rate (MD: −8.97; 95% CI: −11.25, −6.69; *p* < 0.00001; heterogeneity: Chi^2^ = 0.02; I^2^ = 0%; *p* = 0.99, Figure 9 C), and erythrocyte deformation index (MD: −0.33; 95% CI: −0.45, −0.21; *p* < 0.00001; heterogeneity: Chi^2^ = 0.00; I^2^ = 0%; *p* = 0.94, Figure 9 D). Due to heterogeneity in plasma viscosity, the meta-analysis was performed using a random-effects model, and the results indicated that DGMI was effective in reducing plasma viscosity compared to the control group (MD: −0.27; 95% CI: −0.51, −0.02; *p* = 0.003; heterogeneity: Chi^2^ = 12.65; I^2^ = 84%; *p* = 0.002, Figure 9B).

#### Modified Rankin Scale (mRS)

**FIGURE 10:**
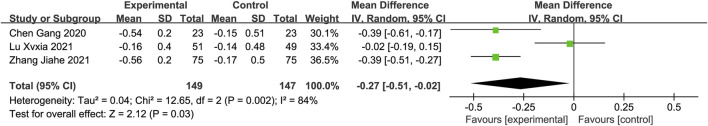
Forest plot of mRS.

Only two studies reported mRS scores (Bao and Peng, 2019; Wang et al. 2024), with 105 participants in the DGMI group and 103 in the control group. Data analysis using a fixed-effects model revealed that DGMI was more effective in reducing mRS than the control group (MD: −0.39; 95% CI: −0.52, −0.25; *p* < 0.00001; heterogeneity: Chi^2^ = 0.77; I^2^ = 0%; *p* = 0.38, Figure 10).

#### Adverse reactions

Only three of the 18 studies analyzed (Luo and Chen, 2019; Hu et al., 2021; Lu, 2021) provided information on adverse reactions, as presented in Table 2. Although the adverse reactions reported in the experimental group were milder and fewer than those in the control group, they did not interfere with the treatment or lead to worse outcomes. This finding suggests the safety of DGMI in combination with edaravone. However, the inclusion of a limited number of articles and the absence of adverse reactions in several studies could introduce bias when assessing treatment safety.

## Discussion

Ischemic stroke, is the second leading cause of death worldwide and accounts for approximately 68% of all cerebrovascular diseases. The prevalence of ischemic stroke is a significant health concern owing to the characteristics of modern lifestyles, such as excessive consumption of oil, sugar, salt, and fat. Clinical research has focused on treating ischemic stroke and implementing primary prevention measures to address unhealthy lifestyles. Current treatment approaches primarily involve anticoagulation and thrombolysis with the aim of restoring blood flow in obstructed blood vessels. Thrombolytic drugs, when administered at specific doses, are considered the optimal treatment because of their ability to restore blood flow without inducing adverse effects, such as bleeding. However, certain medical conditions and patient factors limit the number of individuals who can receive thrombolytic therapy within the recommended timeframe. Consequently, clinicians are exploring alternative treatment options to improve their effectiveness.

In China, numerous clinical trials have demonstrated that combining TCM with Western medicine for the treatment of ischemic stroke can provide additional advantages. These include reducing the occurrence of complications, alleviating symptoms, lowering disability rates, and improving the long-term survival rates. The pathogenesis of acute ischemic stroke is complex and encompasses small hypertensive arteriosclerosis, cerebral atherosclerosis, reduced cerebral blood flow, hypercoagulability, hyper-adhesiveness of the blood, platelet aggregation, and thrombus formation. This intricate mechanism disrupts blood flow and causes neurological deficits in the brain. Infarct lesions in ischemic stroke can be categorized as ischemic foci and ischemic penumbra. During reperfusion in the ischemic penumbra, excess free radicals are generated, potentially resulting in nerve cell damage and apoptosis.

Edaravone is frequently prescribed for patients with acute ischemic stroke who are unable to receive intravenous thrombolytic therapy in time (Edaravone Acute Infarction Study Group, 2003). It functions as a potent hydroxyl radical scavenger and an antioxidant. It rapidly crosses the blood-brain barrier and has various beneficial effects, such as scavenging free radicals, inhibiting lipid peroxidation, and providing protection against apoptosis, necrosis, cytokine-induced damage, and neurovascular injury (Inokuchi et al., 2009; Kikuchi et al., 2009). Diterpene ginkgolide meglumine injection (DGMI) is composed of ginkgolides A, B, and K, which are the primary constituents derived from *Ginkgo bilob*a. According to TCM, these components promote blood circulation, activate blood flow, and resolve blood stasis. From a modern medicine perspective, they can significantly dilate blood vessels in the area affected by cerebral ischemia, reduce vascular resistance, scavenge oxygen free radicals, and affect the concentration and ratio of central neurotransmitters, such as dopamine, dihydroxyphenylacetic acid, and 5-hydroxytryptamine. These neurotransmitters are closely associated with cerebral ischemia and reperfusion (Ma et al., 2012; Zhang et al., 2015; Zhang et al., 2016). However, the mechanism of action of DGMI in brain protection extends beyond its effect on neurotransmitters.

SOD activity decreases and MDA content increases following cerebral ischemia, resulting in alterations in mitochondrial permeability and release of apoptotic factors. Caspases are crucial for apoptosis, and animal experiments have demonstrated that DGMI can reduce caspase activity and prevent neuronal cell apoptosis following cerebral ischemia-reperfusion. This could be one of the mechanisms by which DGMI exerts neuroprotective effects (Yan et al., 2010; Chen et al., 2014). Blood rheological indicators are significant in the pathogenesis of cerebral infarction. The active ingredient in *G. biloba,* diterpene lactone dextran, exhibits platelet-activating factor receptor antagonism, which can prevent platelet activation and inhibit platelet aggregation by antagonizing the PI3K-Akt signaling pathway, reducing thrombus formation, and correcting hemodynamic abnormalities (Liu et al., 2015; Song et al., 2021). NSE is a protein that specifically indicates brain tissue injuries. The detection of NSE can be used to diagnose acute cerebral infarction and evaluate the extent of tissue damage. DGMI combined with edaravone has been shown to significantly reduce NSE concentration (Gelderblom et al., 2013).

In conclusion, the combination of DGMI and edaravone showed therapeutic benefits in patients with acute ischemic stroke. It has been observed to decrease NIHSS scores, improve the Barthel Index and other scales, decrease MDA levels, improve SOD activity, and positively affect blood rheological indices. Furthermore, the combination therapy demonstrated a favorable safety profile. However, it is important to note that the literature included in this study was of low quality, which inevitably introduced bias and affected the overall quality of the evidence. Therefore, more rigorous and high-quality clinical trials are necessary to validate the efficacy of DGMI combined with edaravone.

### Comparison with previous studies

To date, only two meta-analyses on DGMI treatment for cerebral infarction have been published in China. One study analyzed 26 studies involving 2,332 patients (Zhang et al., 2021c). The results of the study showed that the NIHSS scores, Barthel Index scores, and total effective rates were better than those of a single Western medicine. However, this conclusion was based on an analysis of randomized controlled trials that combined DGMI with multiple Western medications. This could potentially introduce bias and compromise the reliability of the conclusions. The other study analyzed data from 9 randomized controlled trials involving 1,129 patients (Jin et al., 2019).The study found that DGMI was effective in treating cerebral infarction and showed positive outcomes in terms of improving NIHSS and ADL scores, indicating its high clinical efficacy. However, the control group in this study was not limited to a specific treatment method and included conventional treatment and other *G. biloba* extracts. Moreover, this study examined both the acute and recovery stages of cerebral infarction. Owing to the presence of confounding variables, definitive conclusions cannot be drawn. Furthermore, neither of these studies analyzed laboratory indicators, and clinical efficacy was the primary outcome measure. The use of different definitions of clinical efficacy can affect the effectiveness of meta-analyses for identifying heterogeneity. Therefore, this study excluded clinical efficacy analysis, focused on objective indicators, such as laboratory parameters, and specifically limited the scope to acute ischemic stroke (AIS) and the drugs DGMI and edaravone. This approach provides more direct evidence for evaluating the efficacy of DGMI and enriches the scope of the study. Additionally, this study manually excluded studies with suspicious or incomplete data to minimize heterogeneity. The research design was clear and focused, and all included studies were randomized controlled trials, thus minimizing bias. Heterogeneity and subgroup analyses were performed after data analysis, making the logical flow more rigorous and the conclusions more reliable.

### Strengths of this study

This study summarizes all relevant clinical studies on the topic and provides a comprehensive evaluation of the efficacy and safety of combining DGMI with edaravone for the treatment of acute ischemic stroke. This study has significant therapeutic implications owing to the limited benefits of Western medicine alone in treating AIS and the observed additional benefits in patients receiving adjuvant TCM treatment. Furthermore, the study’s research objective, robust methodology involving randomized controlled trials, and comprehensive analysis encompassing heterogeneity and subgroup analysis contribute to the reliability of the conclusions.

Another strength of this study is its focus on a highly pure form of TCM extract, DGMI, which exhibits higher purity than other TCM injections. Moreover, the efficacy of each component of DGMI, namely, ginkgolides A, B, and K, is supported by robust evidence and established mechanisms of action (Nabavi et al., 2015; Chen et al., 2018). Recent studies have demonstrated that TCM offers numerous complementary advantages for the treatment of diseases (Wang et al., 2023). This study aligns with the ongoing endeavor to modernize TCM and enhance its integration with Western medicine, thereby offering potential clinical benefits.

### Limitations

However, this study has certain limitations. First, there may be a language bias due to the inclusion of exclusively Chinese-language papers. Second, the quality of the included studies was low as they lacked sufficient descriptions of allocation concealment, blinding, dropped visits, or participant attrition. Third, inconsistencies were observed in the treatment courses, drug dosages, and underlying treatments across the trials. This could potentially result in biased outcomes and contribute to a lack of homogeneity in some outcome indicators. Additionally, the total sample size was relatively small, with only 1,636 cases, all of which were single-center trials, potentially affecting the reliability of the findings. Moreover, the limited generalizability of the conclusions is attributed to the potential influences of the underlying diseases of the patients and the use of single outcome indicators. The long-term outcomes of patients with AIS who receive a combination of the two drugs remain uncertain. Finally, only three studies reported adverse effects, limiting our ability to confirm their relationship with the combination of the two drugs. Further safety evaluations should be conducted using larger sample sizes in future clinical studies.

## Conclusion

In conclusion, available evidence suggests that DGMI can enhance the effectiveness of edaravone in the treatment of patients with acute ischemic stroke. However, the low quality of the included literature introduces bias and necessitates more rigorous and high-quality clinical trials to validate these findings further.

## Data Availability

The original contributions presented in the study are included in the article/Supplementary Materials, further inquiries can be directed to the corresponding author.
